# Inhibition of Quorum Sensing and Biofilm Formation of Esculetin on *Aeromonas Hydrophila*

**DOI:** 10.3389/fmicb.2021.737626

**Published:** 2021-09-24

**Authors:** Bing Sun, Huaizhi Luo, Huan Jiang, Zhennan Wang, Aiqun Jia

**Affiliations:** ^1^School of Environmental and Biological Engineering, Nanjing University of Science and Technology, Nanjing, China; ^2^State Key Laboratory of Marine Resource Utilization in South China Sea, School of Pharmaceutical Sciences, Hainan University, Haikou, China

**Keywords:** quorum sensing, biofilm, esculetin, *Aeromonas hydrophila* SHAe 115, quantitative real-time PCR

## Abstract

Quorum sensing (QS) and biofilm formation inhibition activity of esculetin on *Aeromonas hydrophila* SHAe 115 were evaluated. Exposure to esculetin at 25, 50, and 100μg/ml significantly inhibited the production of protease and hemolysin, the formation of biofilms and attenuated the swarming motility of *A. hydrophila* SHAe 115. Biofilm forming inhibition was also observed through confocal laser scanning microscopy and scanning electron microscope. Quantitative real-time PCR analysis indicated that genes positively related to QS and biofilm formation were downregulated to varying degrees, while gene (*litR*) negatively related to biofilm formation was significantly upregulated. The phenotypic results were in good agreement with gene expression levels. These results indicated that esculetin would be a potential QS inhibitor for *A. hydrophila*.

## Introduction

In the past few decades, indiscriminate use of antibiotics has led to the emergence of multiple drug-resistant bacteria, which has become a major problem threatening the global medical health and public health system (Tanwar et al., 2014). In most cases, due to the way antibiotics kill bacteria or inhibit bacterial growth, selective pressure leads to the emergence of resistant strains. Over the past decade, the development of new antibiotics has declined sharply, while drug-resistant strains have become tougher (Gutiérrez-Barranquero et al., 2015). Therefore, there is an urgent need to develop alternative therapies. These therapies need to target these multi-drug resistant strains in a biofilm state and will not exert selective pressure on resistant strains. The discovery of the bacterial quorum sensing (QS) system provides us with such a promising strategy to prevent and control microbial infections. QS is a cell-to-cell signaling communication system, it involves the production, release, and subsequent detection of chemical signaling molecules, called autoinducers, by a population-density-dependent intercellular communication system allowing bacteria to control the expression of genes related to virulence and pathogenesis. In general, these autoinducers include N-acyl homoserine lactones (AHL) and oligopeptides in gram-negative and gram-positive bacteria, respectively. QS controls the virulence behavior of a broad spectrum of bacterial pathogens and participate in the biofilm formation, a key driver of antibiotic resistance in many infections (Miller and Bassler, 2000; Waters and Bassler, 2005). Hence, QS systems have been proposed as an effective target for antimicrobial therapy, cause it can be blocked by ways of inhibiting the AHL molecule biosynthesis, degrading the synthesized AHL molecules and/or inactivating the AHL receptor protein (Belapurkar et al., 2014).

*Aeromonas hydrophila* is a conditional pathogen that is common to humans, livestock, and aquatic animals (Zhou and Zhou, 2012). *A. hydrophila* can infect molluscs (Grizzle and Brunner, 2009), crustaceans (Ma et al., 2012), fish (Liang and Xie, 2013; Zhao and Wang, 2015), amphibians (Meng et al., 2009), reptiles (Chen et al., 2003), and poultry (Pan et al., 2003). Humans can suffer from diarrhea, food poisoning, and secondary infections due to pathogenic *A. hydrophila* infection (Yang and Wang, 2006). *A. hydrophila* not only poses a threat to human health, but also cause huge economic losses to the aquaculture industry, which has attracted great attention over the world.

Many natural compounds have been reported as QS inhibitors (Jakobsen et al., 2012; Figueroa et al., 2014; Venkadesaperumal et al., 2015), and some of the most effective QS inhibition molecules derived from plants are coumarins, a large structurally diverse family of plant phenolic compounds characterized by their pharmacological properties (Venugopala et al., 2013). Biological studies on coumarins demonstrated that these compounds have potential activities, such as antitumor (Vanamala et al., 2006; Tehsina et al., 2011; Jamier et al., 2014), anti-inflammatory (Fylaktakidou et al., 2004; Bucolo et al., 2009), anticoagulant (Payá et al., 1992; Fereshteh et al., 2014), and antibacterial (Ojala et al., 2000; Souza et al., 2005). Among coumarins, hydroxylated coumarins, such as umbelliferone, daphnetin, and esculetin, showed stronger bioactivities. Wang et al. (2017) demonstrated that esculetin has obvious antibacterial effect on KPC-producing *Klebsiella pneumoniae*. Esculetin also has superior antibacterial activity against the phytopathogen *Ralstonia solanacearum* and can inhibit its biofilm formation (Yang et al., 2016). Thomas et al. (2017) showed that esculetin has a good inhibitory effect on the QS-regulated transcription factor SdiA of *Salmonella typhi* through molecular docking. Recent reports on coumarins inhibiting biofilm formation and reducing virulence factors of *Escherichia coli* and *Pseudomonas aeruginosa* (Lee et al., 2014) have drawn the attention of researchers to the potential of coumarins as QS inhibitors and anti-biofilm agents. According to Duncan et al. (1998), esculetin and umbelliferone could inhibit the growth of *E. coli* O157:H7. Dürig et al. (2010) reported that esculetin was able to prevent biofilm formation of *Staphylococcus aureus* without affecting its cell growth. D’Almeida et al. (2017) conducted a comparison of seven structurally related coumarins (coumarin and different hydroxylated derivatives) on the QS inhibitory and anti-biofilm activities against *P. aeruginosa* and *Chromobacterium violaceum*. The results showed that molecules with hydroxyl groups on the aromatic ring have higher activity on virulence factors inhibition and biofilm formation. Besides, research of Lee et al. (2014) showed that hydroxylation in position C-4 dramatically diminishes the anti-biofilm activity on *E. coli* O157:H7 while hydroxylation in position C-7 enhances it.

According to the above introduction, esculetin has good inhibitory activity against many bacteria, and many plants contain this compound. Our previous phytochemical work also isolated this compound. However, no study on the inhibitory effect of esculetin against *A. hydrophila* has been reported. Hereby, we investigated the influence of esculetin on QS-related virulence factors and biofilm formation of *A. hydrophila* SHAe115. We hope that esculetin can mitigate human disease caused by *A. hydrophila* and/or reduce the loss of aquaculture caused by *A. hydrophila*.

## Materials and Methods

### Bacterial Strain and Culture Conditions

*Aeromonas hydrophila* SHAe 115 used in this study was obtained from China General Microbiological Culture Collection Center. All experiments were conducted at 37°C in Luria-Bertani (LB) medium.

Esculetin was isolated from *Onosma bracteatum* Wall. in our previous study (Sun et al., 2021). It was dissolved in DMSO to prepare a stock solution of 50mg/ml.

### The Minimum Inhibitory Concentration and Growth Measurement

Esculetin was tested against *A. hydrophila* SHAe 115 to determine the minimum inhibitory concentration (MIC) according to Clinical and Laboratory Standards Institute (2015) (Zhou et al., 2017) and with an inoculum of 1–5×10^5^CFUml^−1^. The OD_620_ value of bacterial cultures of *A. hydrophila* SHAe 115 at this concentration was approximately 0.1. Two-fold dilution method was used and a series of diluted esculetin solution (25, 50, 100, 200, 400, and 800μg/ml) was performed in LB broth. The experiment was carried out in 96-well polystyrene microtiter plate, with 10 wells for each concentration. MIC is defined as the minimum concentration of esculetin which inhibited the visible growth of *A. hydrophila* SHAe 115 and sub-MICs were selected for the assessment of anti-virulence and anti-biofilm activity.

For growth measurement, overnight cultures of *A. hydrophila* SHAe 115 were inoculated into fresh LB medium and the optical density (OD) value was adjusted to 0.1 at 620nm. The cultures were transferred to 96-well polystyrene microtiter plate and supplemented with esculetin of different final concentrations (25, 50, and 100μg/ml), and then incubated continuously at 37°C for 24h with shaking (180rpm). DMSO was set as the negative control, and each concentration was set for 10 wells. The bacterial growth was monitored at 1h intervals, and the OD_620_ was recorded by a microplate reader (Biotek, United States).

### Hemolysin Assay

The hemolysin assay was performed with a few changes based on the method of Zhou et al. (2019). 1% overnight culture of *A. hydrophila* SHAe 115 was added to fresh LB medium (OD_620_ ≈0.1) and cultivated in 24-well polystyrene microtiter plate with or without esculetin at 37°C for 24h with shaking (180rpm). The final concentrations of esculetin were 25, 50, and 100μg/ml. DMSO was set as the negative control, and three parallel groups were set for each concentration. After the cultivation, the cultures were centrifuged at 10000rpm for 10min (4°C), and the cell free supernatants were collected. 100μl of cell free supernatants of esculetin treated and untreated cultures were mixed with 900μl of 2% washed sheep blood (in PBS, pH 7.2). The mixture was incubated for 1h at 37°C followed by 10min of centrifugation at 3000rpm. Absorbance of the supernatant was measured at 530nm.

### Azocasein Assay

Azocasein assay was conducted to evaluated the total proteolytic activity of *A. hydrophila* SHAe 115, and the activity was determined by the method of Ding et al. (2018). 75μl of the abovementioned cell free supernatant obtained from each group was added to 125μl of 0.3% azocasein solution (in 50mM Tris-HC1 and 0.5mM CaCl_2_) and incubated at 30°C for 15min. The reaction was ended by adding 600μl of 10% trichloroacetic acid. After centrifugation for 10min at 10000rpm, 700μl of NaOH (1M) was mixed with the supernatant and the OD_440_ was recorded by a microplate reader (Biotek, United States).

### Swarming Motility Assay

The swarming agar was freshly prepared with 0.8% nutrient broth (NB) medium, 0.5% glucose, and 0.3% agar (pH 7.2). 2μl overnight culture of *A. hydrophila* SHAe 115 was inoculated at the center of the agar plate containing a series of esculetin (25, 50, and 100μg/ml). DMSO was set as the control, and three parallel groups were set for each concentration. The plates were cultured at 37°C for 24h, and the swarming migration diameters were recorded (Zhou et al., 2019).

### Biofilm Inhibition Assay

The effect of esculetin at sub-MICs on biofilm formation was measured according to Ding et al. (2018) with some modifications. Briefly, 1% overnight culture of *A. hydrophila* SHAe 115 was added to fresh LB medium (OD_620_ ≈ 0.1) and transferred to a 96-well polystyrene microtiter plate then incubated in the presence and absence of esculetin for 24h at 37°C without shaking. DMSO served as the negative control with 10 wells for each concentration. After the incubation, planktonic cells and spent media were discarded and the biofilms were washed with PBS (pH 7.2) for three times. After being fixed with methanol for 15min, the biofilms were stained with 0.05% crystal violet (CV). Further, excess stain was removed and the biofilms were rinsed three times with PBS (pH 7.2) and bound CV was dissolved with 95% ethanol. Biofilm biomass was quantified by measuring the absorbance of crystal violet-ethanol solutions at 570nm (Biotek, United States).

### Microscopy Analysis

One percent overnight culture of *A. hydrophila* SHAe 115 was added to fresh LB medium (OD_620_ ≈ 0.1), and cultivated in 24-well polystyrene microtiter plate containing glass slides (*d*=14mm) with and without esculetin. Culture was incubated without agitation at 37°C for 24h. DMSO was set as the negative control, and three parallel groups were set for each concentration. After the incubation, planktonic cells and spent media were removed and the glass slides were gently rinsed three times with PBS (pH 7.2).

For scanning electron microscopy (SEM) observation, samples were prepared with the method described by Zhou et al. (2017). Biofilms on the glass slides were fixed with 2.5% glutaraldehyde and dehydrated with graded ethanol (50, 70, 80, 90, and 100%). Subsequently the slides were freeze-dried, gold-coated, and then observed under SEM (Thermoscientific, Verios G4 UC).

Method used in confocal laser scanning microscopy (CLSM) observation was according to Zhou et al. (2018). Briefly, the dried samples were stained with acridine orange (0.1%) for 15min and excess dye was discarded. After being washed with PBS (pH 7.2), the slides were then fixed with paraformaldehyde (4%) for 15min in the dark and subsequently subjected to CLSM (Nikon, A1+ SIM-S). For each group, we randomly selected five areas for image analysis.

### Quantitative Real-Time PCR Analysis

The quantitative real-time PCR (qRT-PCR) assay was carried out under the guidance of Zhou et al. (2018) with slight modification. *A. hydrophila* SHAe 115 was grown in LB medium supplemented with or without esculetin (100μg/ml) at 37°C at 180rpm for 24h. After incubation, cells were washed with sterile PBS (pH 7.2) three times and collected after 10min centrifugation at 4°C. Total RNA was extracted from the bacterial cells using an RNA extraction kit (Biofit Biotechnologies, Chengdu, China) following the manufacturer’s instruction. Reverse transcript reaction was performed with a commercial reverse-transcription enzyme (Tsingke Biotechnology, Beijing, China) according to the manufacturer’s instruction. Quantitative real-time PCR was carried out with an ABI 7300 Plus real-time PCR system. The amplification was carried out in a 20μl reaction volume containing 2×T5 Fast qPCR Mix (SYBR Green I; 10μl, Tsingke Biotechnology, Beijing, China), primers (0.8μl of each), diluted cDNA (1μl), and ddH_2_O (7.4μl). The thermocycling conditions were as follows: incubation for 10min at 95°C, followed by denaturation for 15s at 95°C, annealing and extension at 60°C for 60s. PCR amplification consisting of 45cycles was conducted. All samples were run in triplicate. The primers used in this study were listed in Table 1 (Kozlova et al., 2011). 16S rRNA served as an internal control (Patel et al., 2017). The relative expression of target genes was calculated by the conventional 2^−ΔΔCT^ method proposed by Pfaffl (2001).

**Table 1:** PCR primers for quantitative real-time PCR.

Gene	Primer direction	Sequence (5'−3')	Amplicon size
*litR*	Forward	CATCGAGGTGTTCTCCCGTC	
	Reverse	TCATCCACCAGCTCTTCACG	123
*csgAB*	Forward	TTGTTTCTGGTGGATCTGGATTA	
	Reverse	GGCATTGAGCAGCACGGTA	105
*fleQ*	Forward	ACTTCCCCAACAGCAACTTCA	
	Reverse	CCTTGTCGTGGGTCTGTTGA	126
*fleN*	Forward	CTATGACCGGCTTTTGCAGC	
	Reverse	CCTACGACACCAATCTGCGA	186
*luxS*	Forward	CAGACCCCGAACAAGGACAC	
	Reverse	GCACCGATCAGGCTCATGTA	206
*ahyR*	Forward	TCTTGACGTGATGGGGTTGG	
	Reverse	GGCGGTGATGAACGACAGTA	106
*ahyI*	Forward	CAGATGGGAGGTAGAAAACGAG	
	Reverse	TGGGTATCAGGGGTATCGAAA	123
*16S*	Forward	GCACAAGCGGTGGAGCATGTGG	
	Reverse	CGTGTGTAGCCCTGGTCGTA	299

### Statistical Analysis

All of the experiments were performed in triplicate, and the data were presented as mean±standard deviation (SD). Statistical analysis was carried out with GraphPad Prism 8 software (San Diego, CA, United States). One-way ANOVA plus *post-hoc* Tukey test or two-tail paired *t*-test was used to evaluate the statistical significance between groups. The following terminology is used to denote the statistical significance: ^*^p <0.05, ^**^p <0.01, ^***^p <0.001.

## Results and Discussion

### Determination of MIC and Growth Inhibition Analysis

**Figure 1:**
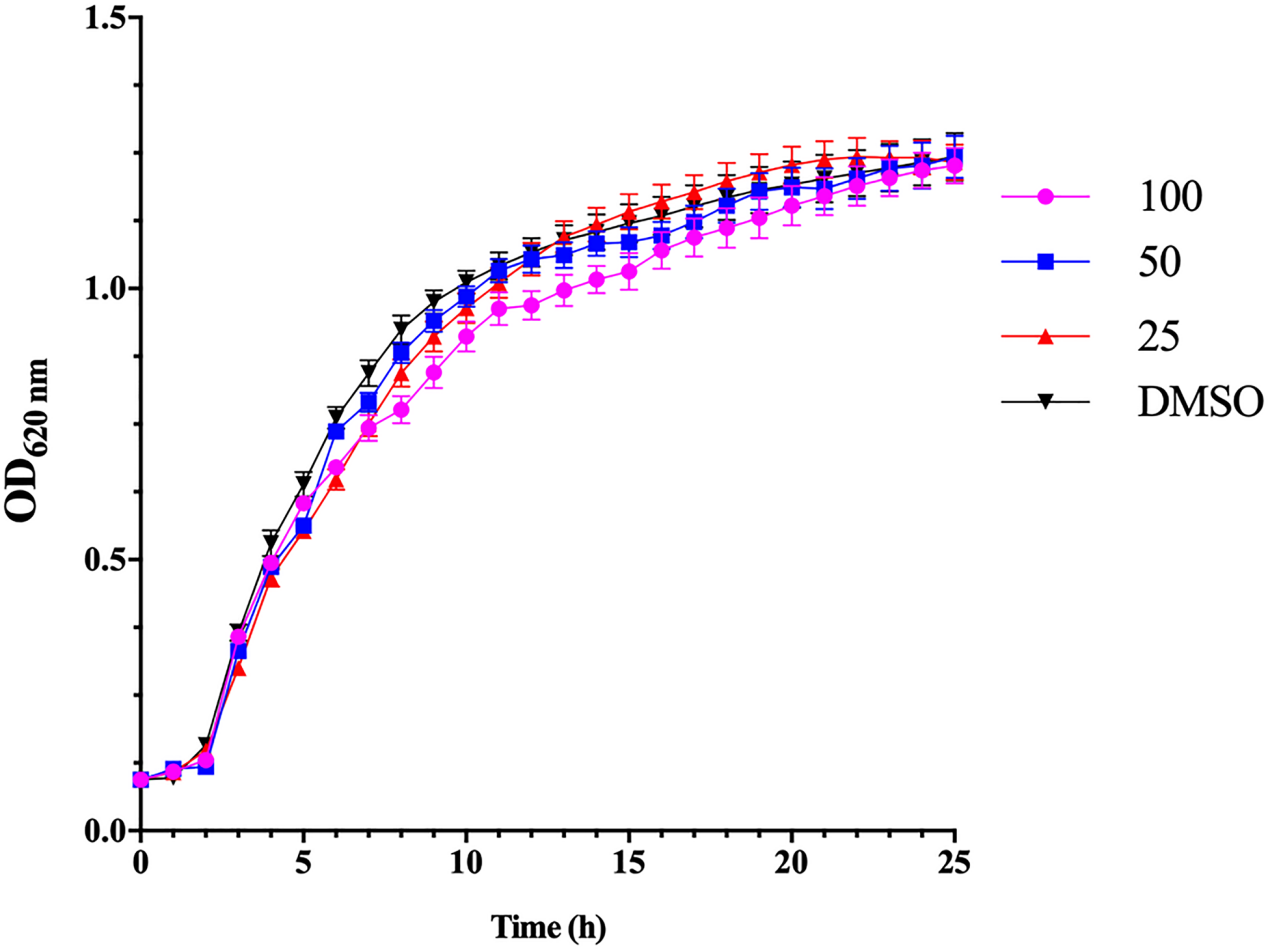
Effect of esculetin at sub-MICs (25, 50, and 100μg/ml) on growth of *Aeromonas hydrophila* SHAe 115. The data represent the mean values of experiments performed in triplicates. Data are presented as the absorbance of mean±*SD*.

The MIC of esculetin evaluated by two-fold dilution was 200μg/ml. All the following experiments were conducted at sub-MICs (25, 50, and 100μg/ml). The bacterial cultures treated with esculetin at sub-MICs did not show any significant inhibitory effect on growth of *A. hydrophila* SHAe 115 compared with the control (Figure 1).

### Inhibition of Hemolysin

**Figure 2:**
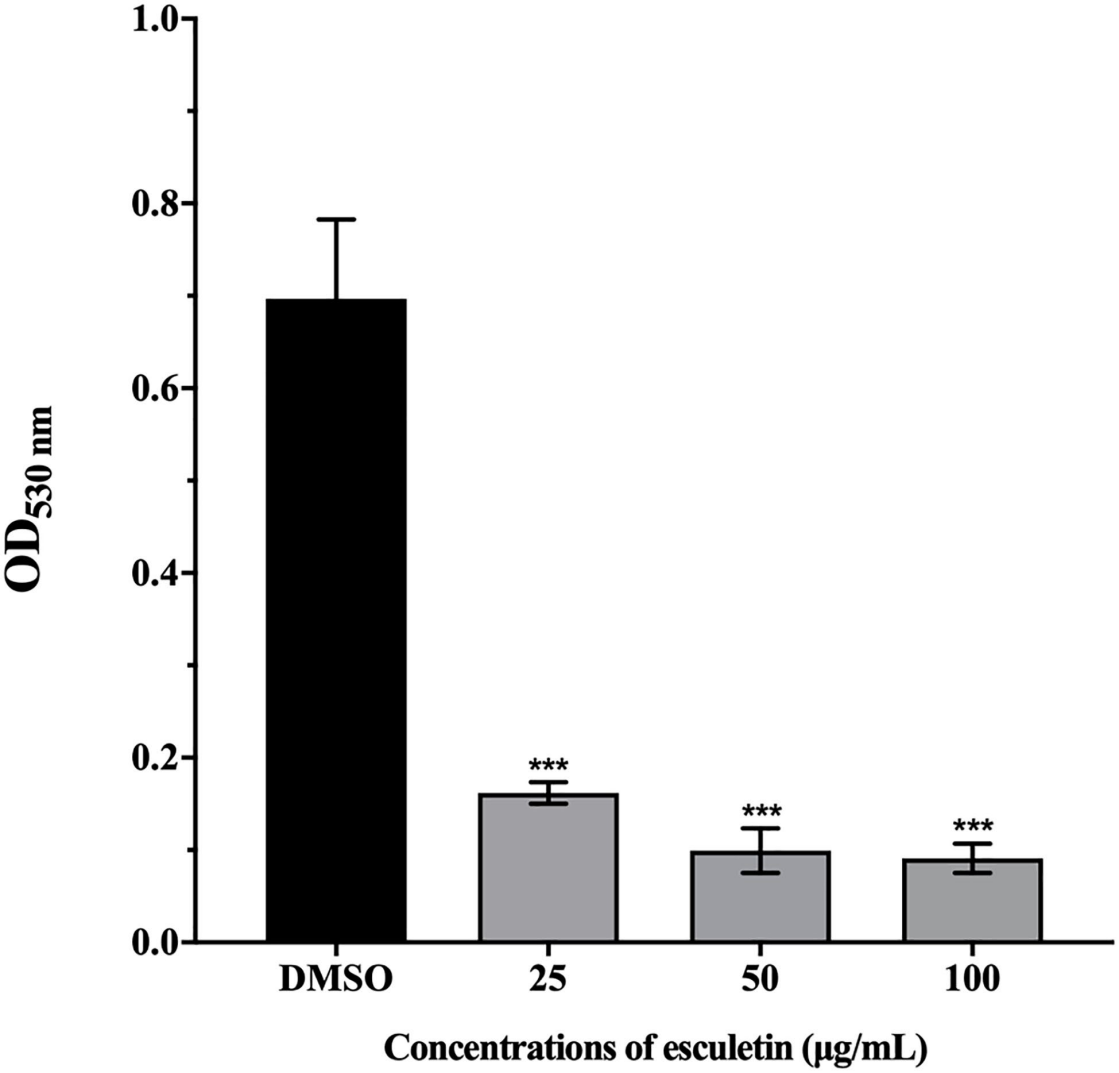
Effect of esculetin at sub-MICs (25, 50, and 100μg/ml) on hemolysin production of *A. hydrophila* SHAe 115. Data are presented as the absorbance of mean±SD of three independent experiments. ^***^p<0.001 compared to the DMSO control group by one-way ANOVA.

As shown in Figure 2, esculetin has a significant inhibitory effect on the production of hemolysin of *A. hydrophila* SHAe 115 at sub-MICs. Compared to the DMSO control group, treatment with esculetin at 25, 50, and 100μg/ml caused reduction in hemolysin production of approximately 77, 86, and 87%, respectively.

Bleeding is a common phenomenon that *A. hydrophila* infects animals, and the hemolytic activity can be detected *in vivo* and *in vitro*. Hemolysin is considered to be the main virulence factor of *A. hydrophila*, so the role of hemolysin is highly valued (James and Trevor, 1988). The hemolysin is a single polypeptide molecule, it is one of the exotoxins (also known as aerolysin) produced by *A. hydrophila*. Allan and Stevenson (1981) injected the exotoxin of *A. hydrophila* on rainbow trout and speckled trout, which caused disease in these fish. Thune et al. (1986) isolated β-hemolysin from a protease-deficient strain of *A. hydrophila* that can kill catfish. Most studies proved that aerolysin genes are highly conserved in *Aeromonas* spp. (Chacón et al., 2003; Lu et al., 2004; Nam and Joh, 2007), indicating that they play an important role in pathogenicity. Our results showed that esculetin significantly inhibited the hemolysin production of *A. hydrophila* SHAe 115, which proved that esculetin can effectively attenuate the pathogenicity of this bacteria.

### Inhibition of Protease Activity

This assay was carried out to analyze the potential of esculetin in inhibiting the production of protease in *A. hydrophila* SHAe 115. Azocasein was used as the substrate. The obtained results indicated that the production of protease was significantly reduced. And the inhibitory ability of esculetin on protease increased with concentration. Protease production was inhibited in the level of 31, 41, and 46%, respectively, in groups supplied with esculetin at concentrations of 25, 50, and 100μg/ml.

**Figure 3:**
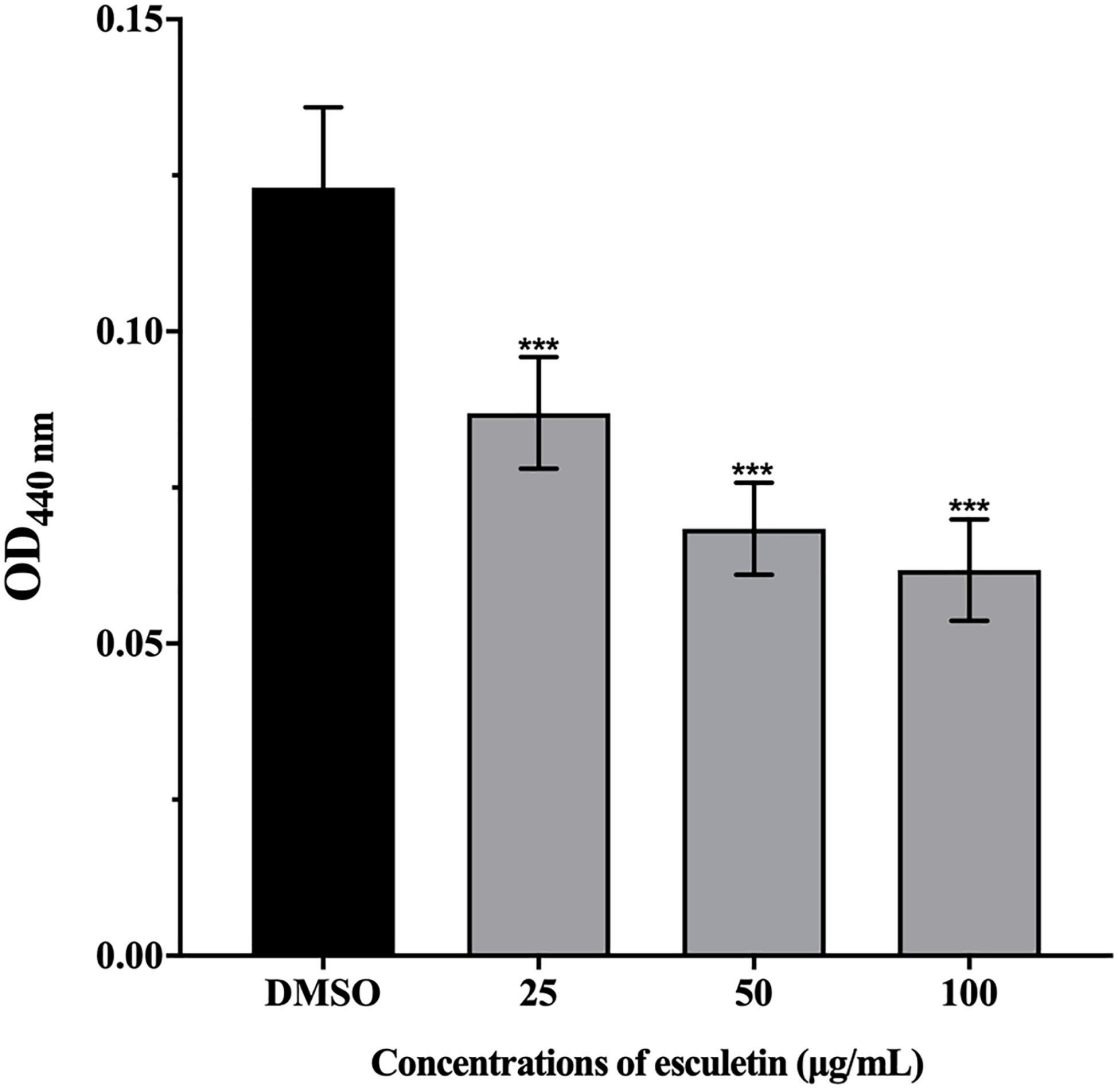
Effect of esculetin at sub-MICs (25, 50, and 100μg/ml) on protease activity of *A. hydrophila* SHAe 115. Data are presented as the absorbance of mean±*SD* of three independent experiments. ^***^p<0.001 compared to the DMSO control group by one-way ANOVA.

Extracellular proteases (Chu and Lu, 2000) are one of the virulence factors of *A. hydrophila*. At present, metalloproteases and serine proteases are widely studied. They are widely present in *A. hydrophila*. Some extracellular proteases have direct pathogenicity, and some can activate other pathogenic factors. For example, the exotoxins secreted by *A. hydrophila* are in the form of an inactive precursor and require extracellular proteases to activate them (Chacón et al., 2003). In our study, as concentration increased, the inhibitory ability of esculetin on proteases gradually increased, as shown in Figure 3. On one hand, the reduction of protease activity can reduce the pathogenicity of *A. hydrophila* SHAe 115; on the other hand, the lack of protease activation of exotoxins (such as hemolysin) will also reduce the pathogenicity of the bacteria.

### Inhibition of Swarming Motility

**Figure 4:**
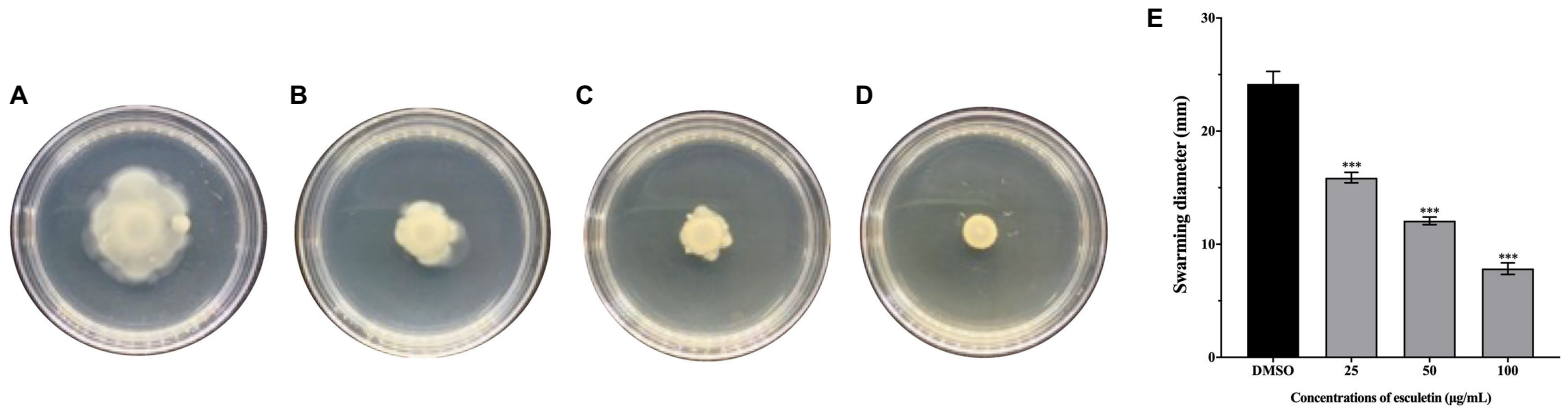
Effect of esculetin at sub-MICs (25, 50, and 100μg/ml) on swarming motility of *A. hydrophila* SHAe 115. **(A)** DMSO, **(B)** 25 μg/ml, **(C)** 50 μg/ml, **(D)** 100μg/ml, **(E)** Swarming zone diameter. Data are presented as the swarming diameter of mean±*SD* of three independent experiments. ^***^p<0.001 compared to the DMSO control group by one-way ANOVA.

The swarming motility of *A. hydrophila* SHAe 115 was clearly visible in DMSO control (Figure 4A). However, on esculetin supplements at concentrations from 25 to 100μg/ml, the swarming motility of *A. hydrophila* SHAe 115 was repressed (Figures 4B–D). And, the diameter of swarming zone decreased as the concentration increased. The bacteria exhibited a total swarming diameter of 24mm. When treated with esculetin at sub-MICs, the swarming diameter decreased to 16, 12, and 8mm, respectively, with an inhibition of 32, 48, and 66% (Figure 4E).

The swarming motility of bacteria has been characterized as flagellar-mediated motility which is regulated by QS system (Köhler et al., 2000; Déziel et al., 2003). And the motility driven by flagellum played an import part in the pathogenicity of the bacteria. Previous studies have suggested that swarming motility can contribute to biofilm formation (Shrout et al., 2010). The swarming motility of *A. hydrophila* SHAe 115 was significantly inhibited by esculetin at sub-MICs (Figure 4). As shown in the figure, the diameters of swarming zone of the bacteria treated with esculetin were much smaller than that of the control, suggesting that esculetin had some inhibitory effects on the swarming motility of the bacteria. And the reason might be the interference of QS system in the pathogen caused by esculetin.

### Inhibition of Biofilm Formation

**Figure 5:**
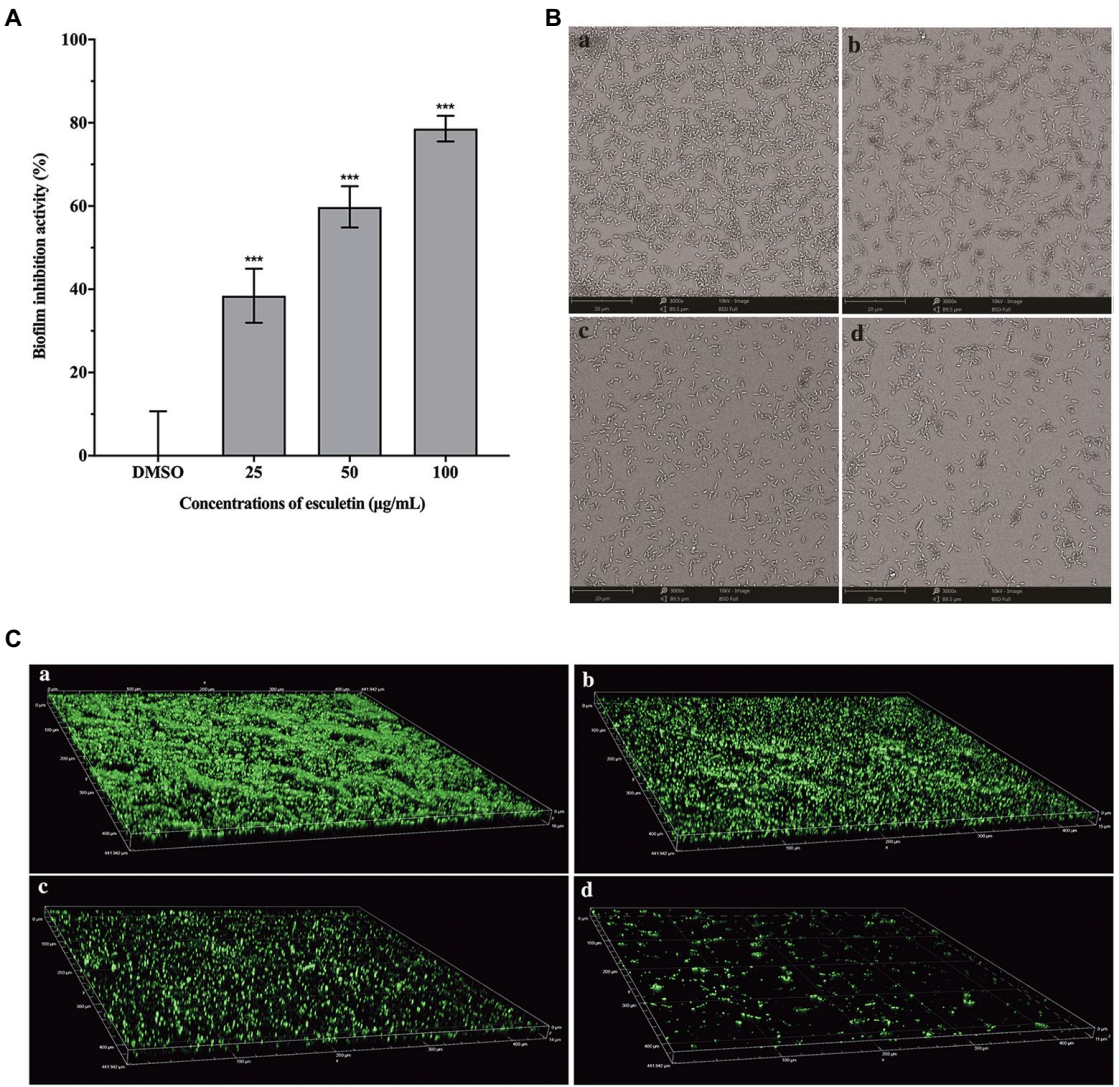
Effect of esculetin on biofilm formation. **(A)** Quantitative analysis of biofilm biomass, **(B)** SEM images, and **(C)** CLSM images of *A. hydrophila* SHAe 115 biofilms treated with **(a)** DMSO, **(b)** 25μg/ml, **(c)** 50μg/ml, and **(d)** 100μg/ml of esculetin. Data are presented as the inhibition rate of mean±SD of three independent experiments. ^***^p<0.001 compared to the DMSO control group by one-way ANOVA.

As shown in Figure 5A, esculetin significantly inhibited the biofilm formation. As the concentration increased, the inhibitory activity of esculetin acted in a concentration-dependent manner. The biofilm inhibition rate was approximately 38, 60, and 79%, respectively, as the bacteria treated with esculetin at 25, 50, and 100μg/ml.

In addition to the quantitative analysis of the biofilm biomass with CV staining method, we also observed the development of biofilm structure through SEM and CLSM after incubation in the presence and absence of esculetin. SEM images showed that the biofilm was thick and dense in the DMSO control group (Figure 5Ba), while with esculetin at sub-MICs treatment, the biofilm was hindered and finally turned sparse as the concentration increased (Figure 5Bb–d). CLSM images also demonstrated similar results. After incubated in the presence of esculetin, the biofilm got sparser with the increased concentration and its thickness reduced from 16 to 11μm (Figures 5Ca–d).

Biofilms are microbial communities, in which bacterial cells are embedded in a self-generated matrix of lipids, exopolysaccharides (EPS), proteins, and nucleic acids that can block the entry of antimicrobial agents into cells (Ramanathan et al., 2017; Zhou et al., 2017). Biofilms are closely related to the multi-drug resistance of many bacteria (Morohoshi et al., 2007). Therefore, destroying or inhibiting the formation of biofilm can be an effective way to attenuate the pathogenicity and drug resistance of bacteria. Our results suggested that esculetin significantly inhibited the biofilm formation of *A. hydrophila* SHAe 115, making it loose and sparse. The possible reason was that esculetin affected the synthesis of the matrix (such as EPS) composing the biofilm.

### Effect of Esculetin on Gene Expression

**Figure 6:**
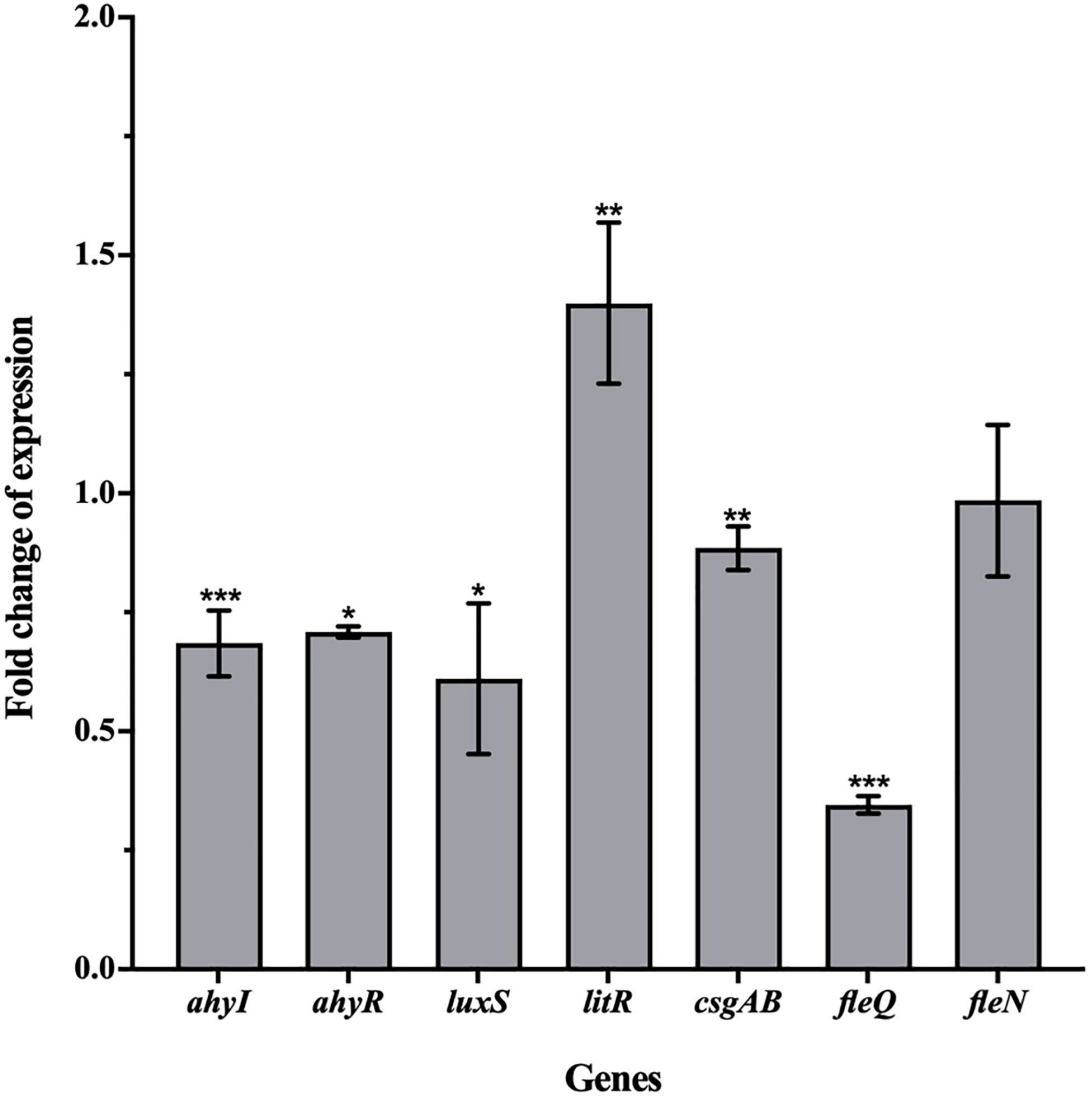
Effect of esculetin at 100μg/ml on the expression of QS-related genes in *A. hydrophila* SHAe 115. Data are presented as the expression fold changes of mean±SD of three independent experiments. ^*^p<0.05, ^**^p<0.01, and ^***^p<0.001 compared to the DMSO control group by *t*-test.

The qRT-PCR assay was carried out to examine the effect of esculetin at 100μg/ml on changes in the gene expression of motility and biofilm formation. The results showed that *ahyI*, *ahyR*, *luxS*, *csgAB*, and *fleQ* were significantly downregulated and their expression levels were reduced by 32, 29, 39, 12, and 65%, respectively. While *litR* was upregulated, the expression level increased by 40%. Esculetin had no obvious effect on *fleN* (Figure 6).

According to the reports of Kozlova et al. (2008 and 2011) and Khajanchi et al. (2009), the *ahyI*/*R* genes in *A. hydrophila* are homologs of *lasI*/*R* that are responsible for regulating the AHL-mediated AI-1 QS system. They demonstrated that the *ahyI*/*R* system was mainly responsible for the QS-related virulence production and biofilm formation in *A. hydrophila*. After treatment with esculetin, the expression levels of *ahyI* and *ahyR* decreased, which interfered the AHL-mediated AI-1 QS system. As a result, the production of virulence factors related to this system and the biofilm formation were inhibited. *litR* gene in *A. hydrophila* is a homolog of *hapR* gene. This gene in *Vibrio cholerae* encodes HapR protein that can negatively regulate bacterial biofilm formation and EPS biosynthesis (Hammer and Bassler, 2003) by lowering the intracellular level of c-di-GMP (Waters et al., 2008). Further, HapR can also negatively affect the transcription of *vpsT*, and the product of *vpsT* (VpsT) is a transcriptional activator required for the expression of EPS biosynthesis operon in *V. cholerae* (Yildiz et al., 2001; Casperlindley and Yildiz, 2004). While in *A. hydrophila*, the homologous gene of *vpsT* is *csgAB* (Kozlova et al., 2011). The increase of *litR* and decrease of *csgAB* gene may affect the biosynthesis of EPS together, thereby affecting the formation of biofilm. FleQ, encoded by *fleQ*, is a master regulator of flagellar gene expression in *P. aeruginosa* (Hickman and Harwood, 2010). FleN, encoded by *fleN*, is an anti-activator of FleQ which downregulates FleQ activity through direct interactions. Kozlova et al. (2011) reported that they found *fleQ* and *fleN* genes in *A. hydrophila*. Hence, the transcription of *fleQ* and *fleN* and interactions between FleQ and FleN play a crucial role in the motility of *A. hydrophila*. Another study of Kozlova et al. (2008) demonstrated that the *luxS* system also existed in the bacteria and it was mainly responsible for the motility. On one hand, the expression level of *fleQ* reduced, which affected the development of bacterial flagella. On the other hand, the expression level of *luxS* also reduced, and the inhibitory effect of esculetin on these two genes may jointly affect the motility of the bacteria.

## Conclusion

The present study explored the inhibitory effect of esculetin on QS-related virulence factors and biofilm formation of *A. hydrophila* SHAe 115. The results showed that esculetin can significantly inhibit the production of hemolysin and protease, affect the swarming motility of *A. hydrophila* SHAe 115 at sub-MICs. They are all the main virulence factors of the bacteria. The formation of biofilm was also inhibited, the biofilm biomass decreased, and the biofilm structure turned thinner and sparser compared to the control group. qRT-PCR analysis indicated that genes positively related to motility and biofilm formation were downregulated to varying degrees, while gene (*litR*) negatively related to biofilm formation was significantly upregulated. The results of swarming motility and biofilm formation were in good agreement with gene expression analysis. Therefore, in combination with the activities against other pathogenic bacteria, esculetin has the potential to be a QS inhibitor.

## Data Availability Statement

The original contributions presented in the study are included in the article/Supplementary Material, further inquiries can be directed to the corresponding author.

## Author Contributions

BS conceived and designed the experiments and also wrote the paper. BS and HL performed the experiments. BS, HL, HJ, and ZW analyzed the data. All authors contributed to the article and approved the submitted version.

## Funding

This work was supported by grants from the National Natural Science Foundation of China (41766006).

## Conflict of Interest

The authors declare that the research was conducted in the absence of any commercial or financial relationships that could be construed as a potential conflict of interest.

## Publisher’s Note

All claims expressed in this article are solely those of the authors and do not necessarily represent those of their affiliated organizations, or those of the publisher, the editors and the reviewers. Any product that may be evaluated in this article, or claim that may be made by its manufacturer, is not guaranteed or endorsed by the publisher.
