# Supplementary figures and images for: Inhibition of Quorum Sensing and Biofilm Formation of Esculetin on Aeromonas Hydrophila
Source: Front Microbiol. 2021 Sep 24;12:737626. doi: 10.3389/fmicb.2021.737626 (PMC8500062; doi:10.3389/fmicb.2021.737626)

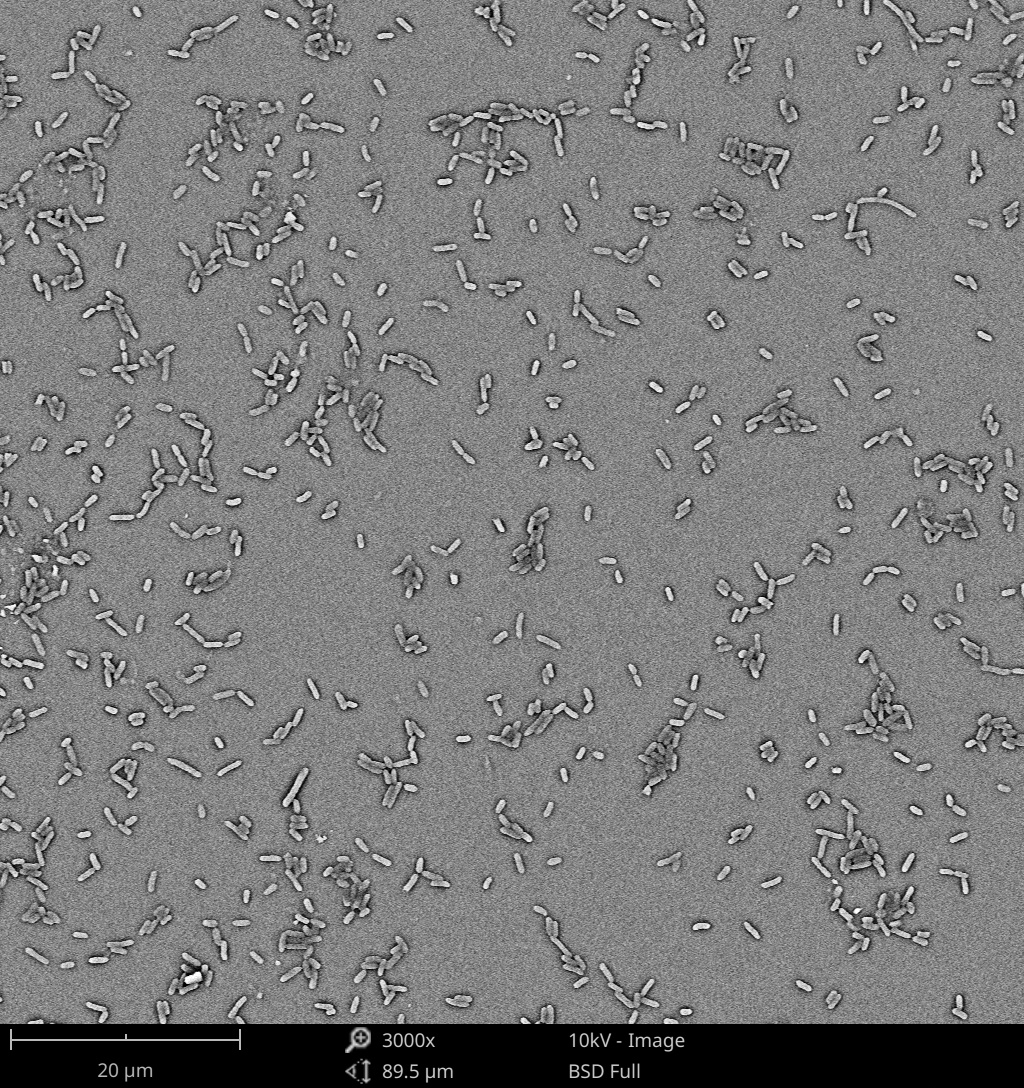

Supplement: Supplementary file 1 [file Data_Sheet_1.ZIP › raw data/SEM/100.jpg]

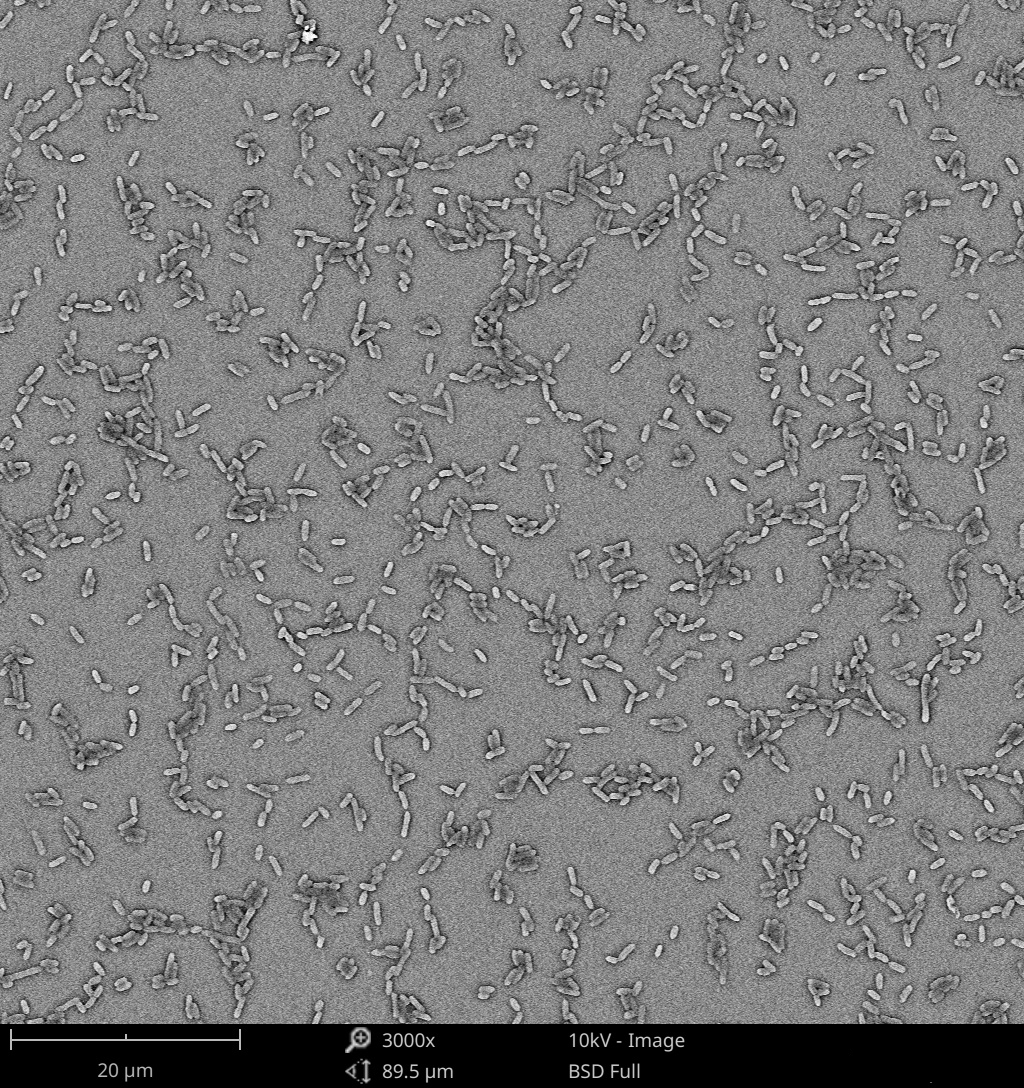

Supplement: Supplementary file 1 [file Data_Sheet_1.ZIP › raw data/SEM/25.jpg]

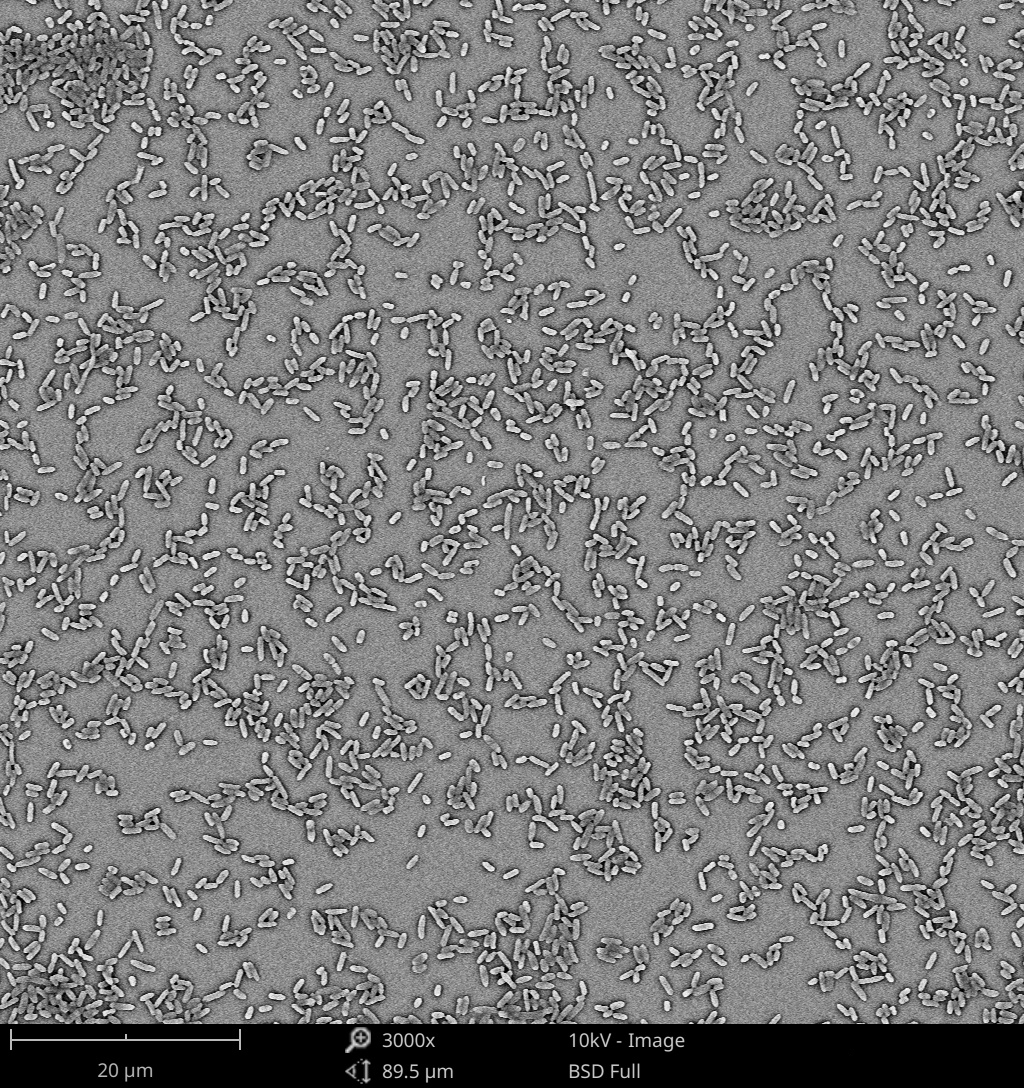

Supplement: Supplementary file 1 [file Data_Sheet_1.ZIP › raw data/SEM/DMSO.jpg]

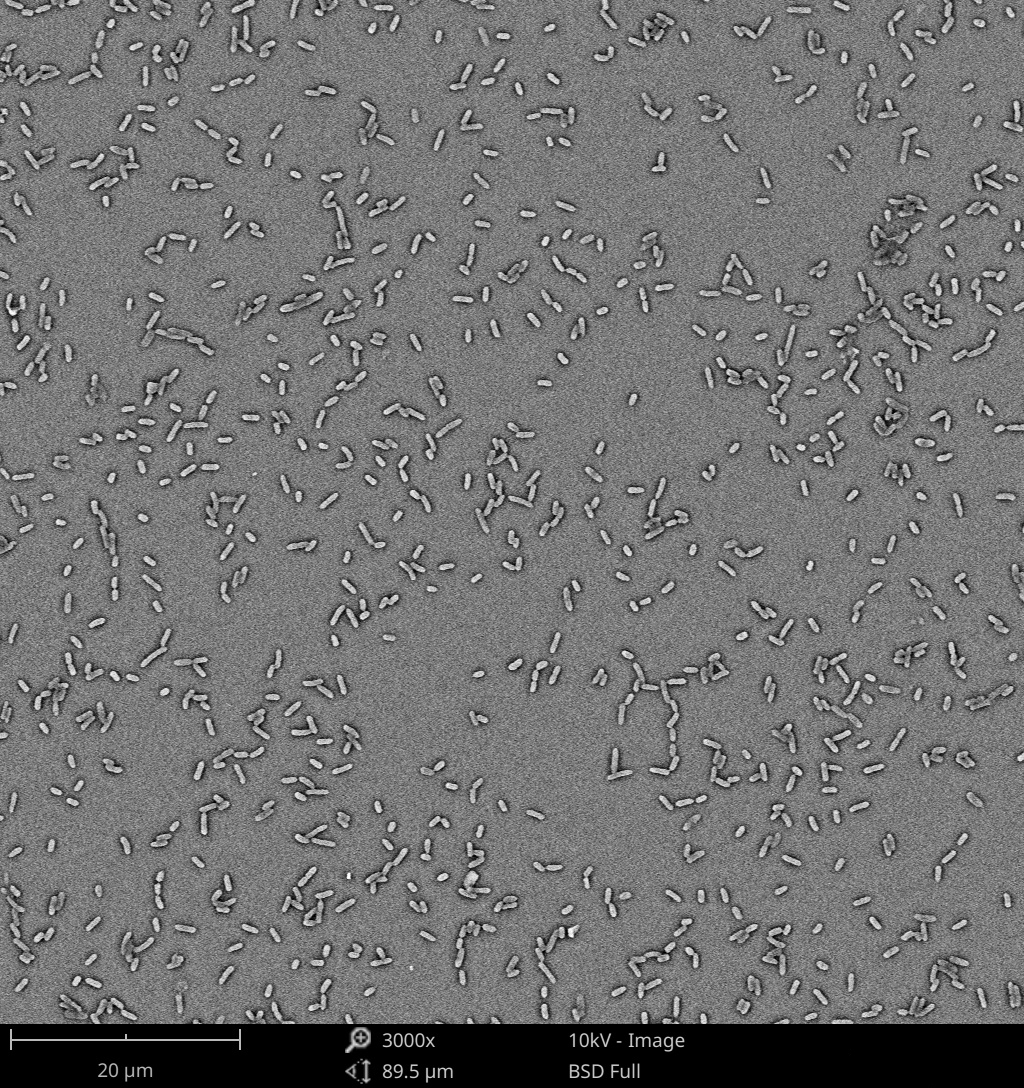

Supplement: Supplementary file 1 [file Data_Sheet_1.ZIP › raw data/SEM/50.jpg]

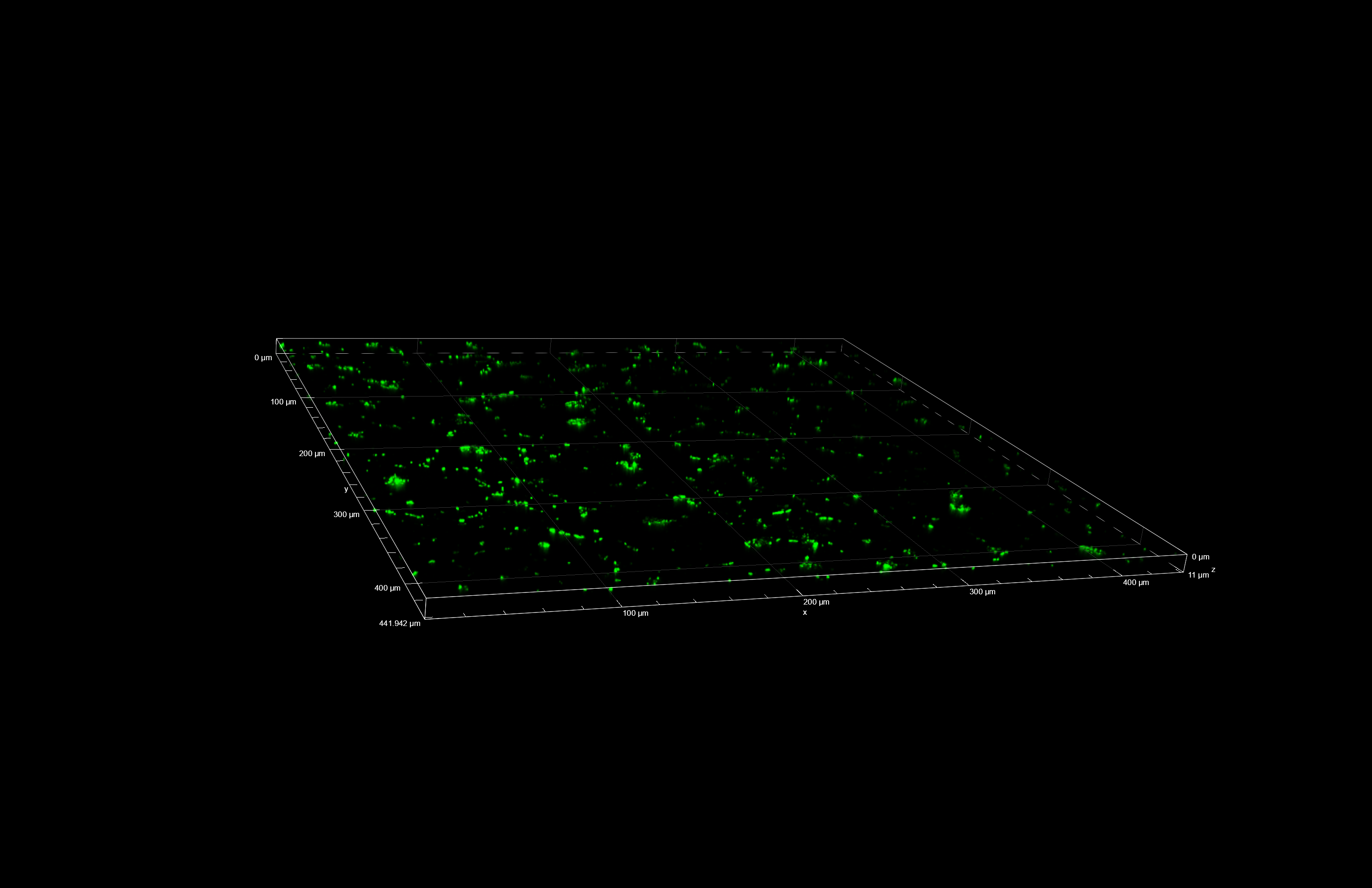

Supplement: Supplementary file 1 [file Data_Sheet_1.ZIP › raw data/CLSM/100.tif]

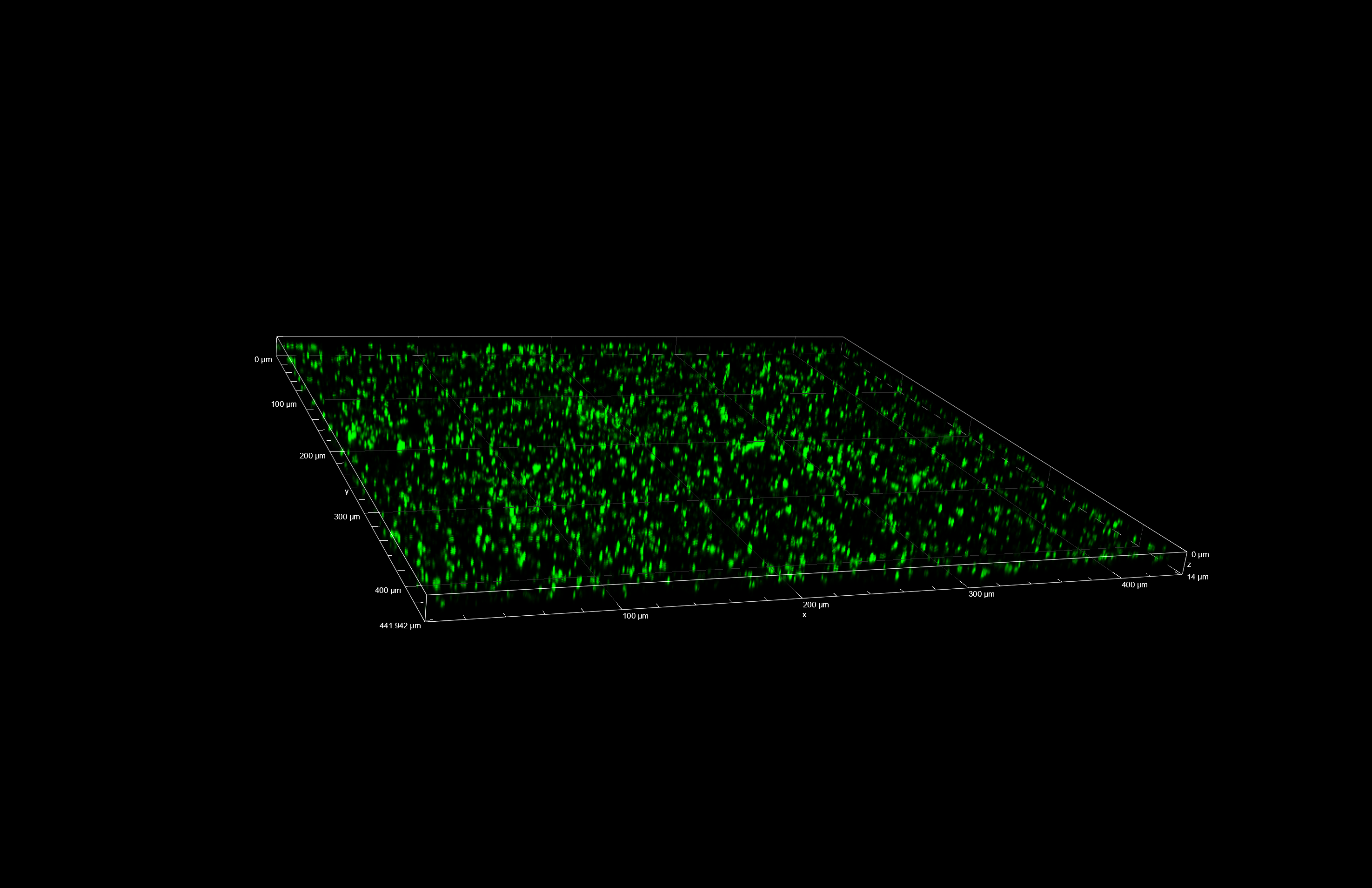

Supplement: Supplementary file 1 [file Data_Sheet_1.ZIP › raw data/CLSM/50.tif]

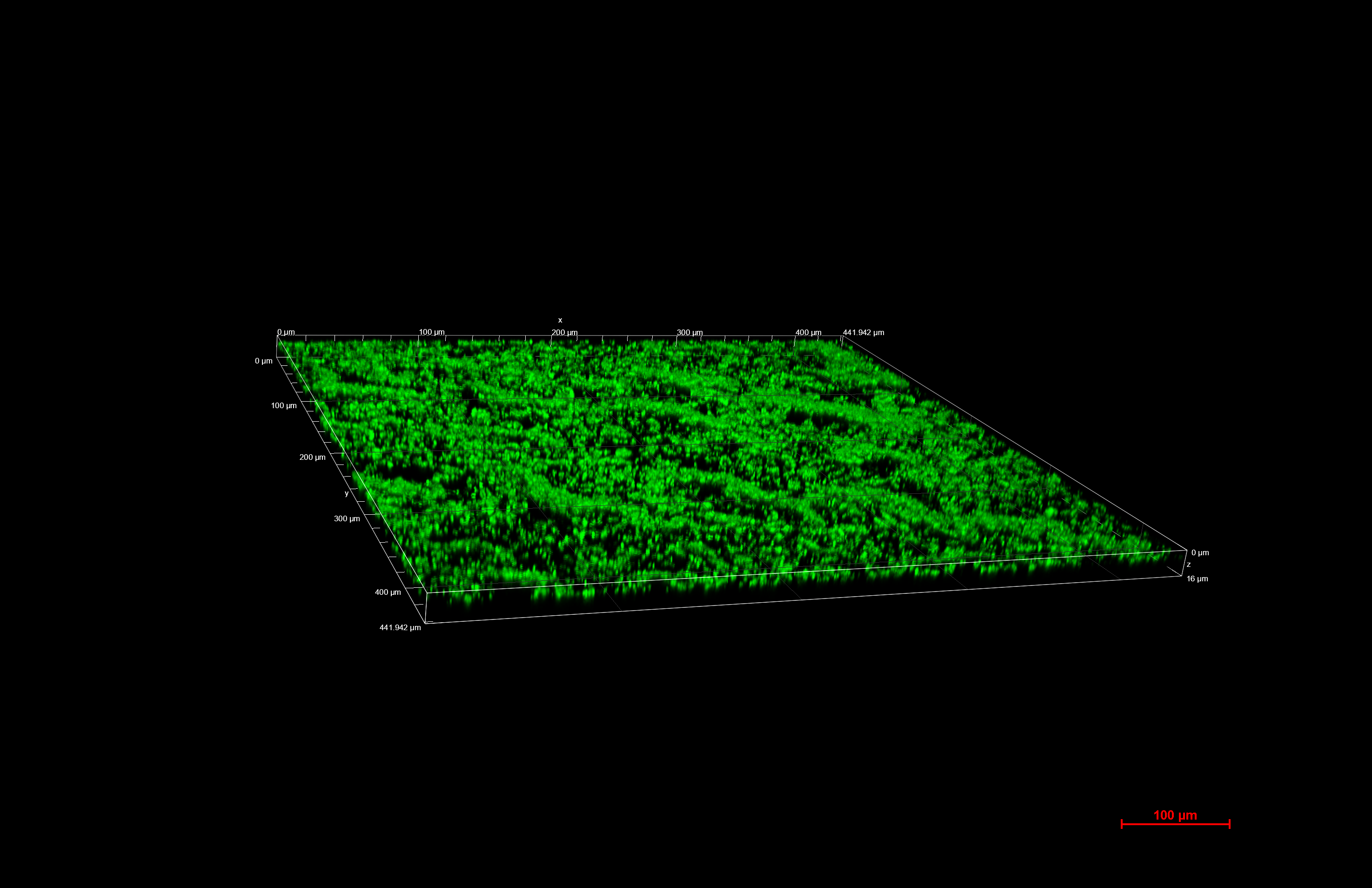

Supplement: Supplementary file 1 [file Data_Sheet_1.ZIP › raw data/CLSM/DMSO.tif]

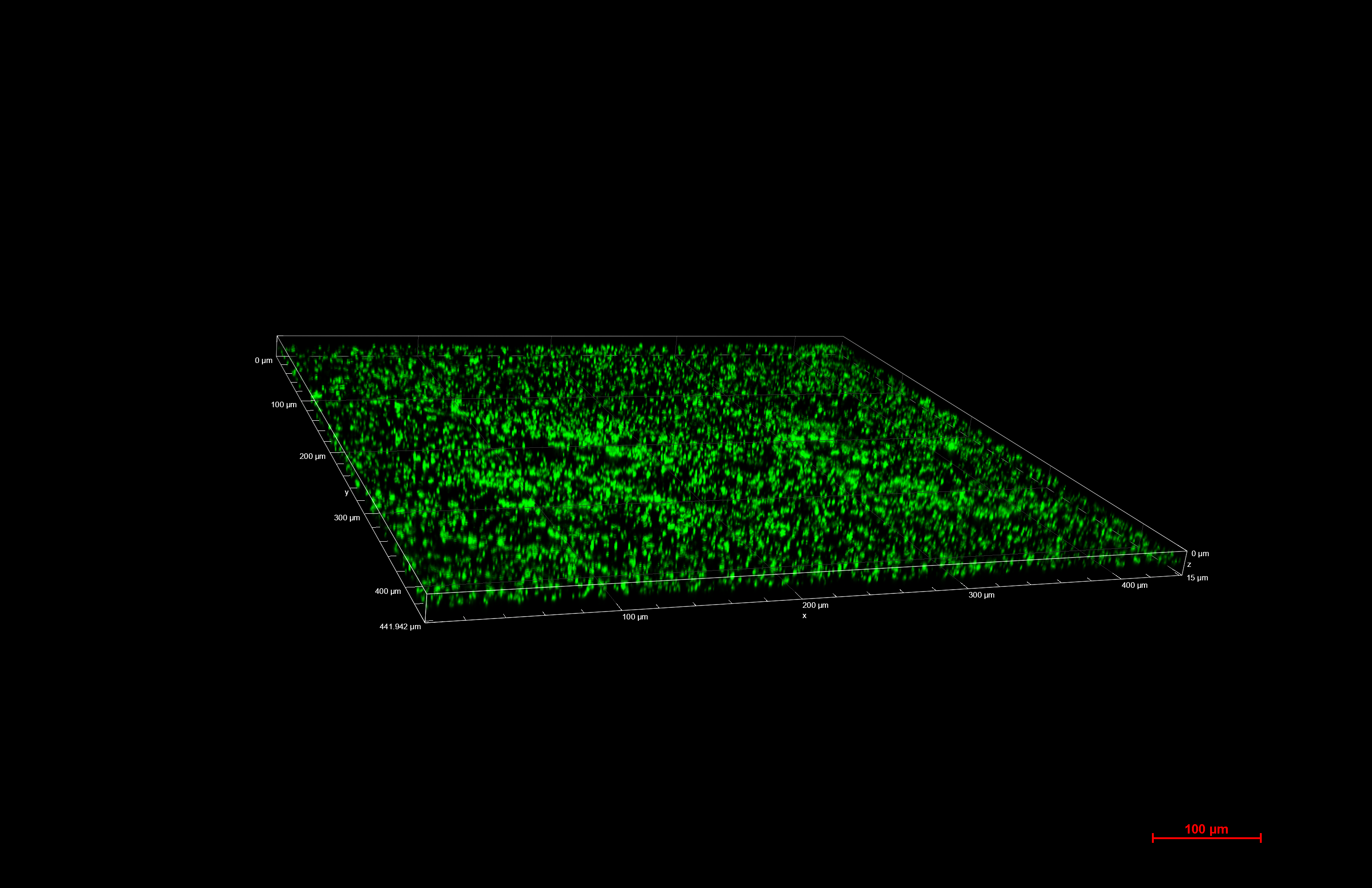

Supplement: Supplementary file 1 [file Data_Sheet_1.ZIP › raw data/CLSM/25.tif]
